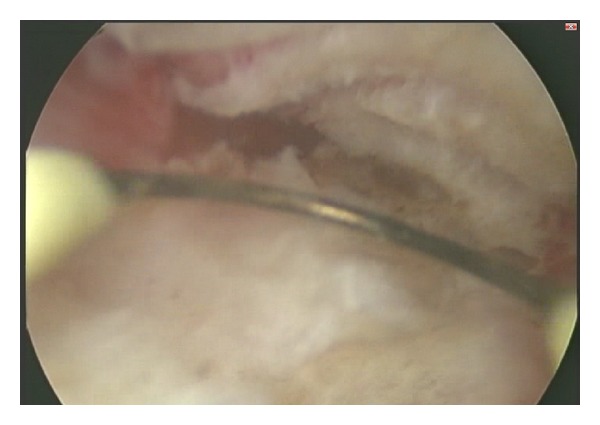# The Diagnosis and Management of Asherman's Syndrome Developed after Cesarean Section and Reproductive Outcome

**DOI:** 10.1155/2013/450658

**Published:** 2013-06-06

**Authors:** Pinar Ozcan Cenksoy, Cem Ficicioglu, Mert Yesiladali, Ozge Kizilkale

**Affiliations:** Department of Obstetrics and Gynecology, Yeditepe University, 34752 Istanbul, Turkey

## Abstract

Intrauterine adhesions (IUAs) frequently occur as a result of trauma to the basal layer of endometrium following pregnancy-related curettage such as incomplete abortion (33,3%), postpartum hemorrhage (37,5%), and elective abortion (8,3%). Hysterotomy, myomectomy, Cesarean section, hysteroscopic procedures, such as resection of submucosal leiomyomata or uterine septae, and endometrial ablation are less common etiologic factors resulting in IUA formation. Patients with Asherman's syndrome usually present with menstrual disturbances, infertility, or recurrent pregnancy loss. A successful treatment of infertility could be achieved by restoration of the uterine cavity, prevention of IUA reformation, and promotion of healing process. We presented the diagnosis and management of a case that suffers from menstrual disturbances and secondary infertility resulted from IUA formation developed after Cesarean section.

## 1. Introduction

Intrauterine adhesions (IUAs) were first described by Fritsch in 1894 and then further studied by gynecologist Asherman [1, 2]. They frequently occur as a result of trauma to the basal layer of endometrium following pregnancy-related curettage such as incomplete abortion (33,3%), postpartum hemorrhage (37,5%), and elective abortion (8,3%) [3]. Basal layer damage leads to partial or complete obliteration of the uterine cavity with surface deficiencies of the endometrium by fibrous bridges between the uterine walls [4]. Patients with Asherman's syndrome usually present with menstrual disturbances such as amenorrhea or hypomenorrhea, infertility, or recurrent pregnancy loss [5]. 

Hysterotomy, myomectomy, Cesarean section, hysteroscopic procedures, such as resection of submucosal leiomyomata or uterine septae, and endometrial ablation are less common etiologic factors resulting in IUA formation. The development of IUA after Cesarean section is uncommon and estimated at approximately 2–2.8% [4]. It may be more likely to result from chorioamnionitis or postpartum endometritis, postpartum curettage, and uterine compression sutures for postpartum haemorrhage [6].

We presented the diagnosis and management of a case that suffers from with menstrual disturbances and secondary infertility resulted from IUA formation developed after Cesarean section. 

## 2. Case History

**Figure 1:**
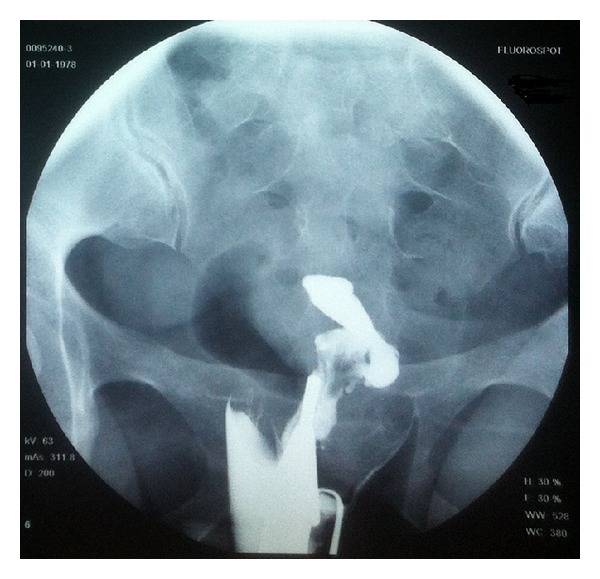


A 34-year-old woman, gravida 1, parity 1, with a previous Caesarean section history of 11 years ago, has applied to Yeditepe University ART Center with secondary amenorrhea and desire for pregnancy. Although she started seeing menstruation again three months after caeserian section, she presented with oligomenorrhea followed by amenorrhea for the last four years. Her vaginal examination and transvaginal USG revealed no abnormalities. Hysterosalphingography (HSG) was planned to evaluate tubal patency and uterine cavity. HSG revealed multiple filling defects in uterine cavity and bilateral tubal occlusion (Figure 1). An operative hysteroscopy was performed to distinguish between varying etiologies of filling defects. Hysteroscopy revealed the extent of endometrial cavity obliteration with dense, multiple adhesions, especially located on isthmus and previous Caesarean incision, and hysteroscopic adhesiolysis was performed (Figure 2). Postoperative intrauterine device and hormonal treatment (4 mg/day estrogen) was applied to prevent the reformation of IUA. A second-look office hysteroscopy was performed to evaluate the endometrial cavity 2 months after the initial procedure. At the second-look hysteroscopy, there was no reformation of adhesions and an adequate uterine cavity was achieved for pregnancy. Patient had resumption of normal menses 3 months after the initial procedure, and IVF treatment was performed 6 months after the initial procedure because of tubal factor. The patient has an ongoing pregnancy. 

## 3. Discussion

IUA would undoubtedly affect the reproductive outcomes but especially if no other etiology for reproductive failure can be found, a successful treatment of infertility could be achieved by restoration of the uterine cavity via lysis of adhesions under direct vision with hysteroscopy, prevention of IUA reformation, promotion of healing process with intrauterine stent and hormonal treatment, and finally performing a follow-up architecture hysteroscopy or hysterosalpingography. On the other hand, even for women who has Asherman's syndrome with additional infertility factors, it is important to restore the uterine cavity and endometrium before IVF treatment. General consensus on the management of Asherman's syndrome exists on the necessity of hysteroscopic adhesiolysis followed by hormone therapy [4]. Particularly in patients with severe Asherman's syndrome, multiple procedures may be required to achieve adequate uterine cavity. Valle and Sciarra reported the postoperative pregnancy rates as 93% in those with minimal disease; term pregnancy rates as 55.6% in patients with severe adhesions and 87.5% in patients with mild adhesions [7]. A recent study found that the severity of intrauterine adhesions before hysteroscopic adhesiolysis affects the conception rates after hysteroscopic adhesiolysis; and the conception rates in women with mild, moderate, and severe adhesions were 64.7%, 53.6%, and 32.5%, respectively [8].

Although Cesarean section is a less common etiologic factor for IUA formation, Asherman's syndrome should be considered in patients with history of Cesarean section presenting with menstrual disturbances such as amenorrhea or hypomenorrhea and infertility, like the present case. To the best of our knowledge, that situation has infrequently been previously reported in the literature. A case reported that woman who presented with dysmenorrhea developed uterine synechiae on the previous uterine incision after previous cesarean deliveries and underwent a total abdominal hysterectomy after failure of a dilation and curettage because of uterine synechiae [9]. Another case presented with secondary infertility showed that severe Asherman's syndrome occurred following a Cesarean section and pregnancy complicated to premature labor and abnormal adherent placentation as placenta increta was achieved through a subsequent intracytoplasmic sperm injection after hysteroscopic adhesiolysis and a cesarean hysterectomy was performed because of placenta increta [10]. A case of a 19-year-old patient who developed secondary amenorrhea after Cesarean section and severe postpartum hemorrhage also demonstrated that severe intrauterine adhesions caused by trauma to the decidua caused by currettage and the decrease in blood flow related to uterine artery ligation because of severe postpartum hemorrhage [11]. 

In the present case, we considered Asherman's syndrome resulting from Cesarean section as an etiological factor for infertility and secondary amenorrhea because of the history of Cesarean section, HSG findings, and the absence of another etiologic factors causing Asherman's syndrome. It is probably related to an adhesive endometrial fibrous process between the uterine walls due to previous Cesarean incision. Because the extent of endometrial cavity obliteration was dense, multiple adhesions, especially located on isthmus and previous Caeserian incision, could be demonstrated by hysteroscopy. We confirmed the diagnosis of IUA formation under direct vision with hysteroscopy which is the gold standard method. Our case was classified as severe Asherman's syndrome according to the modified classification based on the European Society of Hysteroscopy (ESH) and European Society of Gynaecological Endoscopy (ESGE) classification of intrauterine adhesions (1995 version) [12]. We successfully treated the patient with single hysteroscopic adhesiolysis followed by a postoperative hormone therapy. Second-look office hysteroscopy was performed to evaluate the endometrial cavity 2 months after the initial procedure. At the second look hysteroscopy, there was no reformation of adhesions and an adequate uterine cavity was achieved for pregnancy. Patient had resumption of normal menses 3 months after the initial procedure, and an IVF treatment was performed 6 months after the initial procedure because of tubal factor. The patient had an ongoing pregnancy. 

## 4. Conclusion

As the present case, even with no history of chorioamnionitis or postpartum endometritis, postpartum curettage, uterine compression sutures, the probability of Asherman's syndrome should be kept in mind in patients with history of Cesarean section presenting with menstrual disturbances such as amenorrhea or hypomenorrhea and infertility because women with previous cesarean deliveries tend to develop uterine synechiae and that uterine synechiae may have an adverse impact on fertility and the uterine complication rate; therefore, the management of infertility puts forward the evaluation of uterine cavity. Moreover, even if there are additional infertility factors, in the patients with Asherman's syndrome, for the success of IVF procedure it is important to restore the uterine cavity and endometrium. It is also important to realize the high risk involved in such cases during the pregnancy course, and careful perinatal management should be required.

## Figures and Tables

**Figure 2:**